# The Role of Endoscopic Assistance in Surgery for Pediatric Cholesteatoma in Reducing Residual and Recurrent Disease

**DOI:** 10.3390/children11030369

**Published:** 2024-03-20

**Authors:** Nader Nassif, Luca Oscar Redaelli de Zinis

**Affiliations:** 1 Department of Otolaryngology Head and Neck Surgery, Children Hospital, 25100 Brescia, Italy; nadernassif@alice.it; 2 Department of Medical and Surgical Specialties, Radiological Sciences and Public Health, Section of Audiology, University of Brescia, 25100 Brescia, Italy

**Keywords:** cholesteatoma, children, endoscopic surgery

## Abstract

The primary aim of this study was to evaluate long-term recurrent and residual disease after surgery for acquired cholesteatoma in children according to surgical approach. A total of 71 interventions performed on 67 pediatric patients were included in the study. Canal wall-up tympanomastoidectomy (CWUT) was performed in 31 ears (13 with endoscopic assistance), a transcanal esclusive endoscopic approach (TEEA) was used in 22, and canal wall-down tympanomastoidectomy (CWDT) was performed in 18. Overall, the cholesteatoma relapse rate estimated by the Kaplan–Meier method was 47 ± 6% at 12 years; the recurrent cholesteatoma rate was 28 ± 6% and the residual cholesteatoma rate was 26 ± 5%. The relapse rate according to surgical approach was 33 ± 11% for CWDT, 60 ± 9% for CWUT, and 40 ± 11% for TEEA (*p* = 0.04). The difference for recurrent disease was no recurrent disease for CWDT, 42 ± 9% for CWUT, and 32 ± 11% for TEEA (*p* = 0.01). The residual disease rate was significantly reduced with endoscopy: 42 ± 8% without endoscopy vs. 9 ± 5% with (*p* = 0.003). CWDT can still be considered in primary surgery in case of extensive cholesteatomas and small mastoid with poor pneumatization. TEEA can be recommended for small cholesteatoma not extending to the mastoid to reduce morbidity. Endoscopic assistance seems useful to reduce residual disease in CWUT, whereas it does not have a significant impact on preventing recurrent disease.

## 1. Introduction

Cholesteatoma is caused by the squamous epithelium invading the middle ear and temporal bone. Proliferation of the epithelial matrix and continuous keratin production lead to the involvement and destruction of the surrounding structures. It is commonly recognized that pediatric cholesteatoma is more aggressive than adult cholesteatoma because of its rapid growth and expansion [1,2]. Moreover, higher rates of ossicular erosion and more extensive infiltration have been reported compared to adult cases [2]. Likewise, a higher recidivism rate is usually described [3]. This has been correlated not only with more aggressive clinical behavior but also with other factors such as greater mastoid pneumatization, persistent eustachian tube dysfunction in younger children, and overexpression of growth factors that are physiologically produced in childhood [4,5]. The only treatment for cholesteatoma is surgery, with the goals of completely eradicating the disease, achieving a dry and self-cleansing ear, creation of anatomic conditions that prevent recurrence, and preservation of or restoration of hearing. The two classical surgical approaches to treat cholesteatoma are canal wall-down tympanoplasty (CWDT) and canal wall-up tympanoplasty (CWUT). The preferred surgical approach is still a subject of discussion in recent publications [6].

CWDT provides a very large surgical field to remove the disease and offers the best view for postoperative monitoring, allowing for a simpler and prompter recognition of relapsing disease. Moreover, CWDT does not need a second-look procedure or radiologic follow-up, and it is associated with a low risk of relapse. However, there are some drawbacks with CWDT: a protracted recovery interval with prolonged postoperative precautions, a reduction in the volume of the tympanic cavity, with hearing performance that is more likely to be worse than with a normal middle ear volume, and a wider external ear canal that could create aesthetic disturbances and greater problems in fitting a hearing aid. In addition, problems related to water exposure such as thermal stimulation can occur; local care of the open cavity it is often necessary due to a reduced ability to self-cleanse, which causes an accumulation of debris that promotes the growth of granulation tissue and may cause local infections that compromise the quality of life [7]. These disadvantages have been considered particularly disabling in children and the procedure is commonly not recommended in a pediatric age.

In contrast, with CWUT, recovery times are shorter and preoperative external meatus and volume of the tympanic cavity are maintained, with fewer aesthetic and functional disturbances with water exposure and easier fitting if hearing aids are indicated. If necessary, ossicular reconstruction is easier, with better and more predictable hearing performance. On the other hand, the disadvantages of CWUT are dependent on the limited surgical view, which carries an increased risk of residuals, a commonly reported higher incidence of recurrent disease, which imposes a strict observance of follow-up appointments, and the need for a planned second-look procedure or radiologic follow-up [7].

During the last decades, obliteration techniques [8] and endoscopic assistance that aim to improve the results even in children have been developed [9,10]. Endoscopic assistance in middle ear surgery has enhanced the visibility and exposition of the middle ear and mastoid areas. Endoscopes offer a more detailed view, with off-axis visualization enhanced by angled endoscopes. These improvements are particularly helpful in operations for cholesteatoma because of the risk of leaving pathologies in locations hidden to the microscope (e.g., the tympanic recess and supratubal recess), even though the pediatric external ear meatus is narrower than that of the adult. The limitations of endoscopic surgery (single-hand surgery, bi-dimensional view) can be reduced by practical tips (dynamic micro-movements of the endoscope permit spatial awareness of the region of dissection) and technological advancements (curved suction probes and dissecting suction instruments enable axial dissection with angled endoscopes and maintain a blood-free surgical field).

As previously mentioned, anatomical results in terms of relapses are commonly differentiated between recurrent disease and residual disease. Recurrent cholesteatoma develops with the same modalities of primary cholesteatoma (a new cholesteatoma develops into a tympanic retraction pocket or perforation). In contrast, residual cholesteatoma continues to develop after incomplete surgical removal (it grows from small residuals left in place during primary surgery).

The aim of this study was to evaluate long-term recurrent and residual disease after surgery for acquired cholesteatoma in children according to the surgical approach with particular attention to endoscopic assistance. The secondary aim was to analyze the impact on prognosis of other selected variables.

## 2. Materials and Methods

A retrospective study of pediatric patients operated on for primary acquired cholesteatoma between January 2010 and December 2020 was performed. Written informed consent was obtained from all patients and/or parents after a detailed explanation of the surgical procedure and possible risks. This study was approved by the Ethics Committee of our institution. Both authors were involved in the operations. Their experience dates back to the beginning of the 1990s (L.O.R.D.Z.) and the beginning of the 2000s (N.N.).

During this period, three types of approaches were performed: transcanal exclusive endoscopic approach (TEEA), CWUT with and without endoscopic assistance, and CWDT. TEEA consists of cholesteatoma removal through the external ear canal when the endoscope allows for the exposure of the entire lesion through the canal, in which case mastoidectomy is not performed. Rigid endoscopes (Hopkins Telescope, Karl Storz, Tuttlingen, Germany) 3 mm in diameter, 0° and 45°, were routinely used with a three-chip video camera (Karl Storz) and 21-inch high-resolution monitor. 

CWUT and CWDT require a postauricular approach with preservation (canal wall-up) or demolition (canal wall-down) of the posterior wall of the external ear canal. TEEA was reserved for cholesteatomas not extending beyond the lateral semicircular canal. CWUT was reserved for large and cellulate mastoids, whereas CWDT was performed in the case of small mastoids or when major erosion of the posterior–superior aspect of external ear canal wall was present. The surgical steps of CWDT have already been described in a previous report [7]. The ossicular chain was not reconstructed, unless sporadically.

A dedicated database is used in our institution to collect information on patient demographics, origin and extension of the cholesteatoma, surgical approach, ossicular chain condition after cholesteatoma removal, relapses of the cholesteatoma, and timing of relapses. Air and bone conduction thresholds are determined before surgery and during follow-up. Audiograms are performed in a sound isolation booth with a 5 dB step and masking of the opposite ear using narrow band noise by the plateau method. Audiological data are collected according to the Committee of Hearing and Equilibrium criteria. Threshold frequencies of 0.5 kHz, 1 kHz, 2 kHz, and 3 kHz are used. Ossicular reconstruction was performed in a limited group of patients during primary surgery; therefore, reporting standard audiometric results is a source of bias. For the aims of this study, pre- and postoperative bone threshold variability was analyzed to document possible iatrogenic cochlear damage.

All patients were evaluated for disease relapse within two months of surgery, at six months, and every six months thereafter. If the patients were operated by an exclusive endoscopic approach or a closed technique and were clinically free of disease between one and two years after surgery, they were submitted to non-echoplanar diffusion weighted magnetic resonance imaging to rule out residual disease [11]. 

Statistical analyses were performed using the SPSS statistical package (SPSS Inc., Chicago, IL, USA). The cholesteatoma relapse rate (overall, residual, recurrent) was estimated with the Kaplan–Meier method. The entry point was the date of surgery, and the end point was the date of relapse or date of last visit for censored observations. The prognostic value for overall cholesteatoma relapse and separately for recurrent and residual disease of patient, disease, and surgical characteristics (according to STAMCO and Chole classifications) [12,13] was tested by univariate analyses using the log-rank test. The impact of patient age on cholesteatoma relapse was tested with the Mann–Whitney non-parametric test. Statistical significance was defined as *p* < 0.05.

## 3. Results

A total of 71 interventions performed on sixty-seven pediatric patients were included in this study; four patients underwent surgery in both ears at two different times. There were 20 female (34%) and 47 male (66%) patients, with ages ranging from 3 to 16 years (median 9, IQR 7–13). There were 31 right ears (44%) and 40 left ears (56%) affected by the disease. In two patients, facial nerve paralysis allowed for the diagnosis of cholesteatoma; in one patient, an epidural abscess was present. The cholesteatoma developed from pars flaccida in 32 ears and from pars tensa in 39 ears. Cholesteatomas developed from pars flaccida are those originated from the retraction of pars flaccida of the tympanic membrane, whereas cholesteatomas developed from pars tensa are those originated from the retraction of pars tensa of the tympanic membrane. The tympanic cavity was involved in 64 ears, the epitympanum in 62 ears, the mastoid in 38 ears, the sinus tympani in 33 ears, and the supratubal recess in 21 ears.

CWUT was performed in 31 ears (13 with endoscopic-assisted dissection when microscopic exposure was considered inadequate), TEEA in 22 ears, and CWDT in 18 ears.

At the end of cholesteatoma removal, the ossicular chain was intact in nine (13%) ears, the absence of incus was observed in thirty-seven (52%) ears, stapes superstructure was eroded in eighteen (25%) ears, and the malleus handle was absent in seven (10%) ears.

Over a follow-up ranging from 3 to 13 years (median 8 years IQR 6–10), there were 32 cholesteatoma relapses (45%) within 6 years: 18 residual cholesteatomas (25%) and 19 recurrent cholesteatomas (27%) (in five patients with recurrent cholesteatoma there were also residuals). In 19 cases, patients had one relapse; in 12 cases, there were two relapses; and in one patient, there were three relapses. 

None of the patients showed impaired bone conduction threshold at postoperative audiometry.

The median age of patients who relapsed was 8 years (IQR 6.5–12); when comparing it with the median age of non-relapsed patients, which was 11 years (IQR 9–13.5), by Mann–Whitney test, the difference was significant (*p* = 0.005). A significant difference was present also for recurrent (median 7, IQR 5.5–11.5; vs. median 10.5, IQR 8–13; *p* = 0.01) and residual (median 8, IQR 7–10; vs. median 11, IQR 8–13; *p* = 0.02) cholesteatoma. 

The overall cholesteatoma relapse rate estimated by the Kaplan–Meier method was 47 ± 6% at 12 years: the recurrent cholesteatoma rate was 28 ± 6% and the residual cholesteatoma rate was 26 ± 5%. 

Associations between patient, disease, and surgical characteristics were analyzed by the log-rank test (Table 1).

The variables analyzed were sex, side, type of surgical approach, endoscopic-assisted surgery, site of origin, involved sites according to STAMCO [12] and Chole [13] classifications, involvement of the anterior attic, and involvement of sinus timpani (Table 1).

None of the other patient variables were significantly associated with residual or recurrent disease (Table 1).

**Figure 1:**
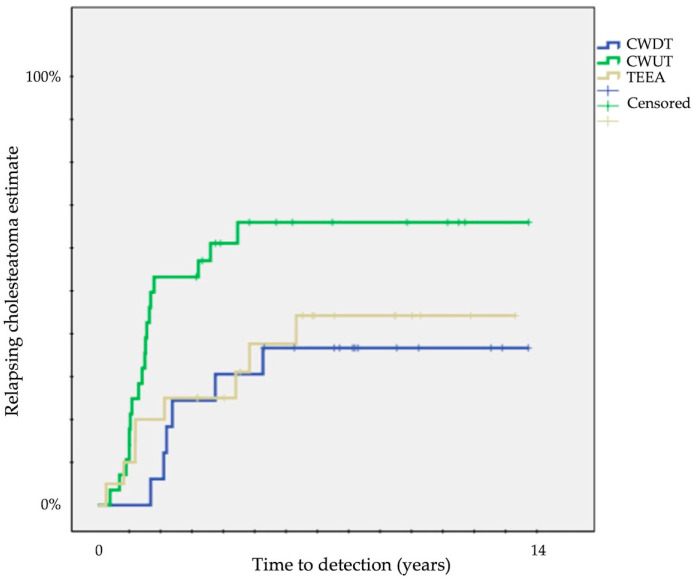
Kaplan–Meier survival curves showing time to detection of overall relapsing cholesteatoma (CWUT, canal wall-up tympanomastoidectomy; CWDT, canal wall-down tympanomastoidectomy; TEEA, transcanal exclusive endoscopic approach).

A significantly different probability of relapse was observed according to the type of surgical approach at 12 years: 33 ± 11% for CWDT, 60 ± 9% for CWUT, and 40 ± 11% for TEEA (*p* = 0.04) (Table 1) (Figure 1). 

**Figure 2:**
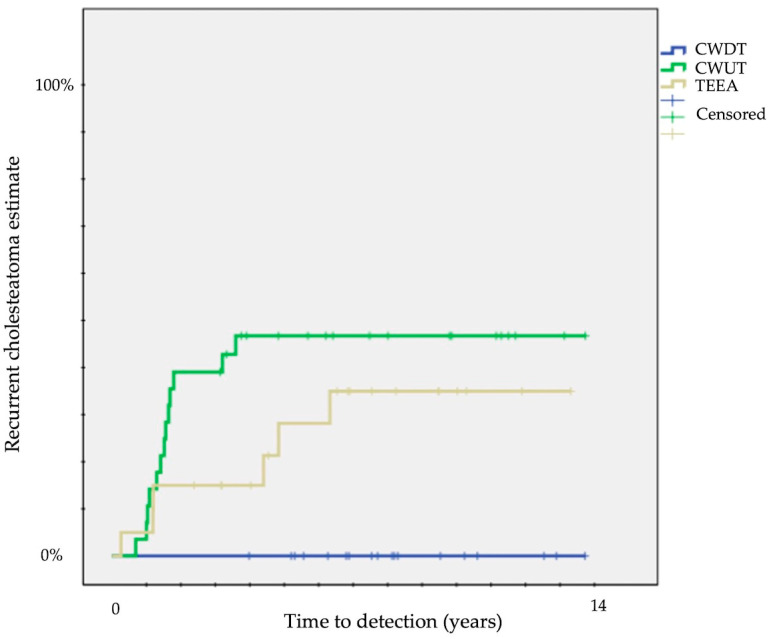
Kaplan–Meier survival curves showing time to detection of recurrent cholesteatoma (CWUT, canal wall-up tympanomastoidectomy; CWDT, canal wall-down tympanomastoidectomy; TEEA, transcanal exclusive endoscopic approach).

The difference was particularly evident for recurrent disease: no recurrent disease for CWDT, 42 ± 9% for CWUT, and 32 ± 11% for TEEA (*p* = 0.01) (Table 1) (Figure 2). 

The probability of residuals was significantly different with endoscopic assistance: 42 ± 8% vs. 9 ± 5% with (*p* = 0.003) (Table 1). 

Among the disease-related variables, only the progressive involvement of ossicular chain according to the STAMCO [12] and Chole [13] classifications was significantly associated with probability of relapse (On, 11 ± 10%; O1, 43 ± 9%; O2, 66 ± 12%; O3, 71 ± 17%; *p* = 0.03) (Table 1). A trend was present only for residuals without reaching statistical significance (On, 11 ± 10%; O1, 25 ± 7%; O2, 32 ± 12%; O3, 43 ± 19%; *p* = 0.6) (Table 1).

**Figure 3:**
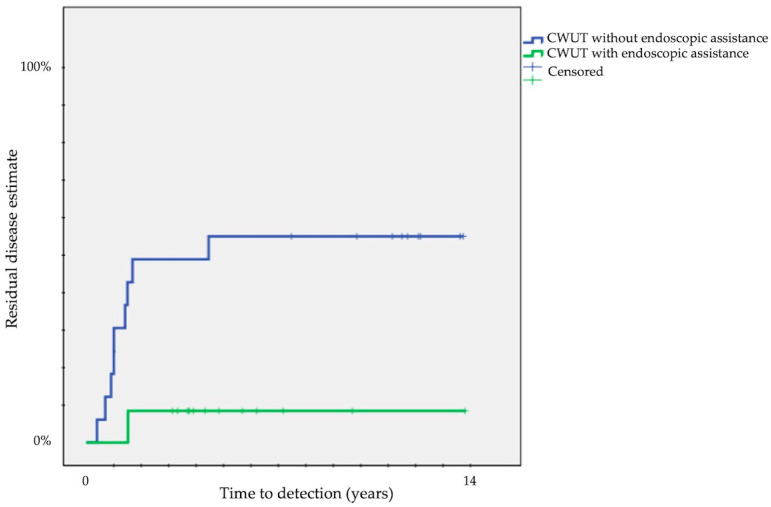
Kaplan–Meier survival curves showing time to detection of residual cholesteatoma according to endoscopic assistance in CWUT (CWUT, canal wall-up tympanomastoidectomy).

Finally, we compared each surgical approach: a significant difference was present comparing residual disease between CWUT (33 ± 9%) and TEEA (9 ± 6%) (*p* < 0.05) and recurrent disease between CWDT (0%) and CWUT (42 ± 9%) (*p* < 0.001) or CWDT (0%) and TEEA (32 ± 11%) (*p* = 0.01). The probability of residuals decreased from 50 ± 12% in CWUT without endoscopic assistance to 8 ± 7% in CWUT with endoscopic assistance (*p* = 0.02) (Figure 3).

## 4. Discussion

The relapse of cholesteatoma in children is still a challenging problem, since values exceeding 50% are still reported in the literature even when follow-up is appropriate [14]. The standard prevalence rate of residual and recurrent disease reported in most of the literature is not the most appropriate to evaluate the occurrence of an event over time, because in the instant when statistical analysis is performed, the follow-up of patients is extremely different, so a survival analysis that gives an estimate of the probability of the event is to be preferred [7,14,15,16].

Our analysis was specifically focused on the occurrence of residual and recurrent cholesteatoma according to surgical approach.

Different surgical techniques to remove cholesteatomas in children and adults are still being discussed in the recent literature, even after endoscopic assistance was added to the surgical armamentarium [3,6,17,18,19,20].

Since 2010, endoscopic surgery has been used to treat cholesteatomas in children in our department. When the cholesteatoma only involved the tympanic cavity and the attic, TEEA was performed. When the cholesteatoma extended beyond the lateral semicircular canal, a postauricular approach was used to perform CWUT or CWDT depending on mastoid pneumatization. CWUT was endoscopic assisted when the surgeon was not sure of complete removal. According to these indications, we analyzed our results to find prognostic factors for cholesteatoma relapses estimated by the Kaplan–Meier method with the log-rank test at 12 years for most of the categorical variables (Table 1), while the prognostic value of the continuous variable “age” was tested by the Mann–Whitney test.

The first piece of information on cholesteatoma relapse according to surgical approach that we gathered from our patients was that only CWDT was associated with lower rates of overall relapsing disease (Figure 1) and with no recurrent disease (Figure 2). 

Anatomical results of CWUT vs. CWDT in children are still a matter of discussion ainderetained [3,6,8,20]. Piras et al. [3] reported 23% of recurrent disease and 21% of residual disease in CWUT vs. 2% of recurrent disease and 8% of residual disease in CWDT. Wang et al. [21] reported a significant higher risk (3.614; 95% CI 1.422, 9.187) of recidivism for CWUT. Kroon et al. [8] reported contrasting results when mastoid obliteration was involved: no recurrent disease and 14% of residual disease in CWUT vs. 21% of recurrent disease and 28% of residual disease in CWDT. Solis-Pazmino et al. [6] in a meta-analysis observed no significant differences in recurrent disease and residual disease between CWUT and CWDT (respectively, cumulative 20% vs. 8% of recurrent disease and 21% vs. 10% of residual disease).

The disadvantages of CWDT can be even greater in children: childhood growth factors cause growth of tissues, thus reducing the caliber of the new external ear canal, causing the loss of self-cleaning and excessive bone regrowth that is irregularly distributed in the mastoid cavity, favoring the development of granulation tissue, debris accumulation, and infection. The presence of a small mastoid that is scarcely pneumatized, complete exenteration of mastoid cells, and partial obliteration of the cavity with postauricular connective tissue can minimize these effects [7]. In our patients, the most important disadvantage were more frequent follow-up visits to remove debris during initial follow-up. The timing for cleaning open cavities was every 6 months in most cases until the patients became adults. Then, the timing of follow-up visits tended to be like that in patients operated in adult age and many of them did not need regular cleansing.

In the last decade, endoscopic assistance or TEEA for acquired children cholesteatoma has been frequently applied even if the results are controversial (Table 2) [16,22,23,24,25,26,27,28,29,30,31,32,33]. Two meta-analyses have been conducted on this topic [9,10]. 

Comparing the results of different authors (Table 2) is difficult: some studies also include congenital cholesteatomas, some only include middle ear cholesteatomas, some compare middle ear with mastoid cholesteatomas; follow-up is extremely variable and in most studies is too short, methods of identification of residual cholesteatoma are frequently insufficient, some authors base the identification on second-look surgery which is only performed in a limited number of ears, and some only analyze residual cholesteatomas (Table 2).

In summary, indications related to endoscopic assistance gathered from the literature are that there is an improvement in the visualization of difficult middle ear recesses. The advantages in avoiding residual and recurrent disease are not universally accepted, but the most accepted advantage of TEEA is to reduce surgical invasiveness, morbidity, and costs of a postauricular approach in cholesteatoma only involving the middle ear and attic [16,22,23,24,25,26,27,28,29,30,31,32,33]. 

According to our experience, residuals were strongly reduced in TEEA or when CWUT was performed with endoscopic assistance (50 ± 12% vs. 8 ± 7%) (Figure 3) and recurrent disease was less frequent in TEEA than in CWUT (43 ± 9% vs. 32 ± 11%). The pathogenesis of recurrent cholesteatoma is probably not related to the surgical approach and technique. However, limited extension of primary disease and preservation of uninvolved mucosa, which are more common when TEEA is performed, could be considered a favorable prognostic factor to avoid recurrent disease. 

We also observed a higher probability of residuals in CWDT. CWDT was never endoscopically assisted in our group of patients, but we are beginning to use endoscopy in CWDT to determine whether it will help to reduce residuals.

The second aim of our study was to analyze the influence of other patients and disease variables on prognosis.

Among the patient variables, the only observation was that children who relapsed were significantly younger (median 8 years, IQR 6.5–12; vs. 11 years, IQR 9–13.5; *p* = 0.005). A significant difference was present for both recurrent (median 7, IQR 5.5–11.5; vs. median 10.5, IQR 8–13; *p* = 0.01) and residual (median 8, IQR 7–10; vs. median 11, IQR 8–13; *p* = 0.02) cholesteatoma. The literature agrees that the rate of cholesteatoma relapse is higher in the pediatric population than in adult patients [1,14], although a specific analysis in children is difficult to extrapolate and it was possible to find significant differences according to age in only a few reports [14,15], but not in other recent studies [6,21].

Among the disease variables that we analyzed, only ossicular chain erosion was associated with some of the different forms of relapses (Table 1).

As was reported by Wang et al. [21], the pathological status of ossicular chain according to the STAMCO [12] and Chole [13] classifications may be useful for the prediction of cholesteatoma recidivism. An explanation for this observation could be related to the higher aggressiveness of a lesion that invades and damages the ossicles.

The most important limitation of the present study is the relatively small sample, which could have been the cause of the lack of significance of some variables, making it nearly impossible to perform multivariable analysis. The second limitation is that we did not report audiometric results in a standard manner but only in terms of bone conduction, because the ossicular chain had not been reconstructed in most cases during surgical intervention. A third limitation could be that surgical experience, usually considered among the prognostic factors of ear surgery, was not analyzed, given that both surgeons participated in all operations.

## 5. Conclusions

Children have a high risk of developing cholesteatoma relapses and long-term follow-up is required. CWDT can still be considered the most effective approach to prevent relapses and can be proposed in primary surgery in case of extensive cholesteatomas and small mastoids with poor pneumatization. TEEA can be recommended for small cholesteatoma not extending to the mastoid to reduce morbidity. Endoscopic assistance seems useful for reducing residual disease in CWUT, whereas it does not have a significant impact on preventing recurrent disease. The role of endoscopic assistance in CWDT needs further analysis.

However, further research on a larger number of patients is needed to support our experience.

## Figures and Tables

**Table 1:** Cholesteatoma relapse estimates (Kaplan–Meier analysis of survival, log-rank test).

Variable	(No of Patients)	Overall Relapse Estimate (%)	Time (Years)	*p* Value	Residual Disease Estimate (%)	Time (Years)	*p* Value	Recurrent Disease Estimate (%)	Time (Years)	*p* Value
**Site of origin**	pars flaccida (32)	51 ± 10	10	0.8	28 ± 9	12	0.9	30 ± 9	12	0.7
	pars tensa/sinus (39)	44 ± 8	12		26 ± 10	12		27 ± 7	12	
**Sex**	female (21)	49 ± 11	10	0.9	24 ± 9	11	0.8	31 ± 11	12	0.9
	male (50)	47 ± 7	12		27 ± 7	12		27 ± 7	12	
**Side**	right (31)	41 ± 9	12	0.4	23 ± 8	12	0.7	27 ± 8	12	0.9
	left (40)	52 ± 8	12		29 ± 7	12		29 ± 8	12	
**Surgical approach**	CWD (18)	33 ± 11	12	0.04	33 ± 11	12	0.1	0	12	0.006
	CWU (31)	60 ± 9	12		34 ± 9	12		43 ± 9	12	
	endoscopic (22)	40 ± 11	12		9 ± 6	12		32 ± 11	12	
**Endoscopic assistance**	no (36)	50 ± 8	12	0.6	42 ± 8	12	0.003	19 ± 7	12	0.1
	yes (35)	46 ± 10	12		9 ± 5	12		40 ± 10	12	
**Difficult sites (STAMCO)**	no (26)	51 ± 11	12	0.6	30 ± 10	12	0.9	29 ± 9	12	0.7
	supratubal recess (12)	43 ± 15	11		21 ± 13	11		17 ± 11	11	
	sinus tympani (24)	38 ± 10	12		21 ± 8	12		26 ± 9	12	
	both sites (9)	67 ± 16	12		33 ± 16	12		44 ± 17	12	
**Tympanic involvement (STAMCO)**	no (7)	33 ± 19	9	0.3	17 ± 15	9	0.4	17 ± 15	9	0.4
	yes (64)	49 ± 7	12		27 ± 6	12		30 ± 6	12	
**Attic involvement (STAMCO)**	no (9)	48 ± 18	11	0.7	22 ± 14	11	0.8	39 ± 18	11	0.7
	yes (62)	47 ± 7	12		27 ± 6	12		27 ± 6	12	
**Supratubal recess involvement**	no (50)	45 ± 7	12	0.5	25 ± 6	12	0.8	28 ± 7	12	0.8
	yes (21)	53 ± 11	12		29 ± 10	12		29 ± 10	12	
**Sinus tympani involvement**	no (38)	49 ± 9	12	0.9	29 ± 8	12	0.9	25 ± 7	12	0.7
	yes (33)	46 ± 9	12		24 ± 8	12		31 ± 8	12	
**Mastoid involvement (STAMCO)**	no (33)	35 ± 9	12	0.1	18 ± 7	12	0.2	23 ± 8	12	0.4
	yes (38)	57 ± 8	12		33 ± 8	12		32 ± 8	12	
**Ossicular condition (STAMCO)**	intact (9)	11 ± 11	9	0.03	11 ± 11	9	0.6	0	9	0.2
	M + S+ (37)	43 ± 9	12		25 ± 7	12		29 ± 8	12	
	M + S− (18)	66 ± 12	12		32 ± 12	12		40 ± 12	12	
	M− (7)	71 ± 17	12		43 ± 19	12		31 ± 19	12	
**Ch (Chole classification)**	1a (3)	33 ± 8	7	0.9	33 ± 27	7	0.7	33 ± 8	7	0.8
	1b (3)	100	5		0	6		0	5	
	2a (5)	61 ± 10	3		40 ± 8	3		40 ± 22	7	
	2b (25)	47 ± 13	12		20 ± 8	12		20 ± 8	12	
	3 (35)	40 ± 22	12		30 ± 8	12		30 ± 8	12	
**Mastoid status**	well pneumatized (27)	51 ± 10	12	0.4	19 ± 8	12	0.5	36 ± 10	12	0.3
	partially pneumatized (22)	50 ± 11	12		32 ± 10	12		32 ± 10	12	
	sclerotic (22)	38 ± 11	12		28 ± 10	12		14 ± 7	12	

Legend: CWD, canal wall-down; CWU, canal wall-up; STAMCO, STAMCO classification; M, malleus handle; S, stapes superstructure.

**Table 2:** Summary of clinical characteristics of recent reports of treatment of cholesteatoma in children with endoscopic assistance.

Author	Cholesteatoma Type	Surgical Approach (No of Ears)	Residual Cholesteatoma Rate	Recurrent Cholesteatoma Rate	Mean Follow-Up Months (Range)	Notes
**Marchioni et al. [21]**	Including congenital	MA CWUT (28)	17%	34%	36 (8–88)	Second look in selected cases
		TEEA (31)	13%	19%		
**Hunter et al. [22]**		MA CWUT (47)	9%	9%	18.8 (7–48)	Second look in selected cases
		MA + EA (21) TEEA (8)	10%	10%		
**James et al. [15]**	Including congenital, ear canal, and implantation	MA CWUT (108)	24%	Not analyzed	Median length of maximum follow-up 74 months	Second look in selected cases
		MA CWUT + EA or TEEA (127)	15%		Median length of maximum follow-up 38 months	
**Sarcu et al. [23]**	Including congenital	MA CWUT + EA (42)	14%	Not analyzed	60.2 (12–188)	In 17% of ears, residuals were not detected with microscope but were detected with endoscope during initial surgery; second look in selected cases
**Cohen et al. [24]**	Including congenital	MA CWUT (24)	25%	Not analyzed	Not reported	Second look in all cases
		MA CWUT + EA or TEEA (32)	28%			
**Ghadersohi et al. [25]**	Including congenital	MA + EI (7)	29%	14%	31 (9–55)	Second look in selected cases MRI in all cases
		EA (middle ear) + MA (mastoid) (9)	7%	13%		
		TEEA (22)	0	5%		
**Le Nobel 2017 et al. [26]**	Exclusive middle ear/attic including congenital	MA atticotomy + EA (79)	9%	Not analyzed	52 (12–126)	Second look or MRI in selected cases; residual correlated with intraoperative bleeding
		TEEA (33)	12%			
**Glikson et al. [27]**	Exclusive middle ear/attic	MA CWUT (19)	16%	37%	37.2	Clinical and MRI follow-up
		TEEA (30)	10%	7%	32.6	
**Yaniv et al. [28]**		MA CWUT (42)	38%	14%	51	Clinical and MRI follow-up
		MA CWUT + EA (49)	18%	33%	64	
**Dixon et al. [29]**	Exclusive middle ear/attic	MA (112)	11%	Not analyzed	Not reported	Two years’ second look or MRI in selected cases
		TEEA (65)	6%			
**Curran et al. [30]**		MA (30) or MA + EA (35)	5%	2%	(24–60)	Including adults; 18 months’ second look or MRI in all cases
		TEEA (26)	4%	4%		
**Manzoor et al. [31]**	Including congenital	MA (253)	6%	4%	Not reported	Including adults; second look in 28% of cases
		MA + EA (79) or TEEA (43)	13%	7%		
**Hu et al. [32]**	Extended to the mastoid, including congenital	MA CWUT + EI (32)	6%	9%	24 all patients	In 1 ear, residuals were not detected with microscope but were detected with endoscope during initial surgery; CT +/− MRI to detect residual disease

Legend: MA, microscopic assistance; CWUT, canal wall-up tympanoplasty; TEEA, transcanal exclusive endoscopic approach; EI, endoscopic inspection; CT, computed tomography; MRI, magnetic resonance imaging.

## Data Availability

The data presented in this study are available on request from the corresponding author. Data contained within this article are not available due to privacy issues.
